# Different MicroRNAs expression in *Mycobacterium tuberculosis* and correlation with prognosis of the disease

**DOI:** 10.1080/15476286.2025.2609681

**Published:** 2025-12-28

**Authors:** Karthikeyan Sundaram, Sridhar Rathinam

**Affiliations:** a Department of Herbal Pharmacology and Environmental Sustainability, Chettinad Hospital and Research Institute, Chettinad Academy of Research and Education, Kelambakkam, Chennai, Tamilnadu, India; b Chettinad Hospital and Research Institute, Chettinad Academy of Research and Education, Kelambakkam, Chennai, Tamilnadu, India

**Keywords:** Tuberculosis, miRNA, mycobacteria, circular-miRNA, disease progression

## Abstract

Tuberculosis, caused by *Mycobacterium tuberculosis*, is an infectious disease linked to high mortality and can stay in the host cell longer when inactive. Multiple factors are linked to disease prognosis, including microRNAs. It is a diminutive single-stranded RNA that regulates the expression of its target mRNAs. It consists of a brief nucleotide sequence, often 19–25 nucleotides in length, of non-coding RNA. It is also essential for early embryonic development, invasion, cell migration, apoptosis, and cell death. The review aims to analyse the transcriptome characteristics of various miRNAs in the tuberculosis prognosis. However, *miR-155, miR-29*, circ-miRNA, and lncRNAs regulate gene expression. In TB patients’ serum exosomes, *miRNA-146* expression was noticeably higher than in healthy individuals. Drug-resistant tuberculosis was related to *miR-548 m, miR-631, miR-328-3p*, and *miR-let-7e-5p*, as well as *let-7b-5p, miR-30a-3p, IL-27*, and *CXCL9/10/11* in TB patients’ lesion tissue and peripheral blood. Therefore, further miRNA research will focus on TB progression.

## 1. Introduction

Tuberculosis (TB) is an infectious disease caused by *Mycobacterium tuberculosis* (MTB). MTB can stay in the host cell longer while it is dormant, even though it is a preventable and treatable infection. The WHO Global Tuberculosis Report 2024 revealed that 10.8 million individuals (95% UI: 10.1–11.7 million) became ill with TB globally in 2023 [1]. The progression of TB from latent to the active state depends on the host’s primary defence mechanism. Although, the MTB gene’s function is capable of adapting within the alveolar macrophage, and enhances their self-defence mechanism against autophagosome-lysosome fusion action [2]. Differently expressed (DE) circRNA, miRNA, and mRNA control transcription and immunological responses in TB. They usually affect transcription, protein binding, and immunological responses positively and negatively. A recent study identified a core ceRNA regulatory network with five circRNAs (*hsa-circ_0001844, hsa-circ_0000566, hsa-circ_0007587, hsa-circ_0086710*, and *hsa-circ_0005408*) and one miRNA (*hsa-miR-607*) that compete to regulate IFNG, three mRNAs, including *GBP2*, impact *IFNG* simultaneously. More research is needed to address microbe-host interaction gaps. Database for Annotation, Visualization, and Integrated Discovery (DAVID) analyzes DE genes. CircRNAs are ribonuclease and exonuclease-resistant due to their covalently closed-ring structure and lack of a 5′ terminal cap and 3′ terminal poly(A) tail. CircRNAs’ long half-life and stability make them promising biomarkers [3–5]. Notably, miRNAs affect a number of important processes, such as apoptosis, differentiation, and cell proliferation. Most significantly, abnormal patterns of miRNA expression have been linked to the pathophysiology of numerous diseases. These patterns control the generation of numerous cytokines and chemokines that modify the host immune response throughout different disease states. Importantly, transcriptome patterns in whole blood can detect TB, infection, and illness using biomarkers including IFN-γ, IP-10, LAM, and IL-2 [6]. Also, the limitation behind the conventional diagnosis of TB including insensitivity of microscopy and the time required for culture make regular TB testing challenging. Molecular accelerators like GeneXpert are expensive and uncommon in rural regions. The molecular testing or culture of smear-negative pulmonary and extra-pulmonary TB needs invasive techniques. Therefore, this review aims to examine the different types of miRNAs, and other regulatory genes involved along with mRNA to the expression of genes in modulating the MTB survival as dormant in macrophages as well as progressing their ability to the active stage.

## 2. Role of miRNAs

MiRNAs, single-stranded, non-coding RNA with a 19–25 nucleotide sequence, govern the expression of their target mRNAs. They are essential for invasion, cell migration, apoptosis, cell death, and early embryonic development. Also, persistent miRNAs are discovered in sputum, and several diseases are significantly linked to their differential expression. In severe asthma, sputum *miR-629-3p, miR-223-3p*, and *miR-142-3p* levels are higher, indicating neutrophilic airway inflammation. Sputum miRNAs contribute to asthma’s inflammatory phenotype. NSCLC sputum had higher *miR-223* levels than healthy controls [7–9].

### 2.1. Role of miRNA-155 in TB pathogenesis

**Figure 1.**
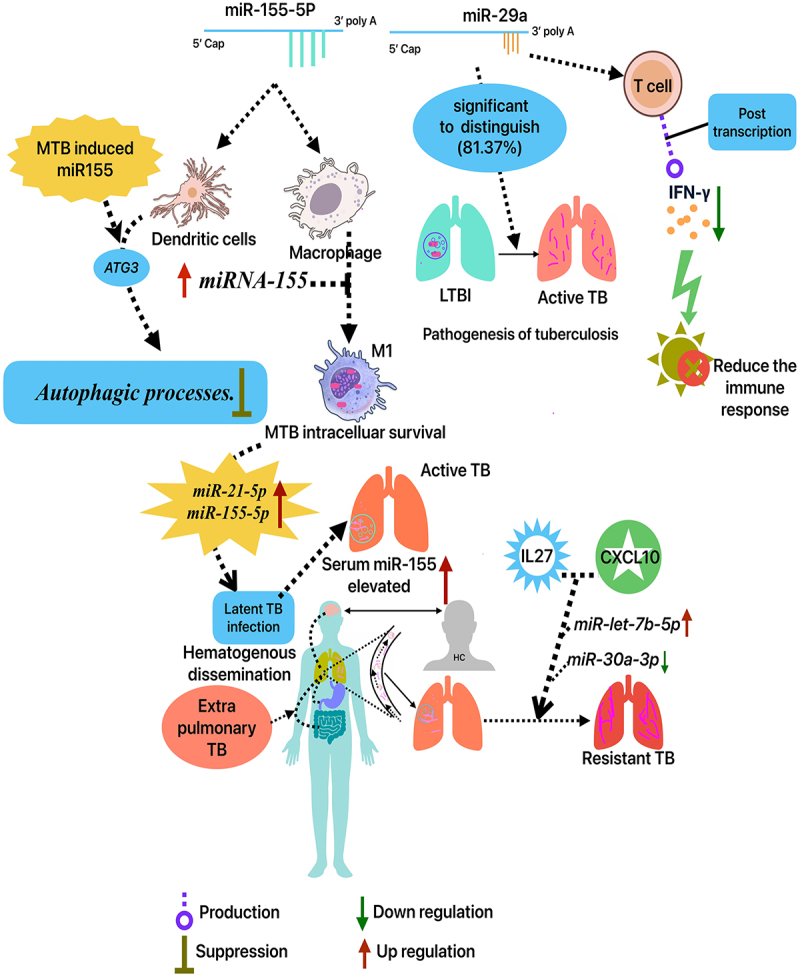
Different miRNAs associated with the progression of TB.

The *miRNA-155* is a master regulator for numerous diseases particularly this miR-155 is linked to pathogenesis of TB infection. Through the regulation of Ras Homologue Enriched in Brain (RHEB) gene expression, it was found that *miRNA-155* may promote cellular autophagy and facilitate the elimination of MTB from the host. While Akt, AMP-activated protein kinase, and glycogen synthase kinase-3β affect RHEB activity, the mechanisms governing RHEB expression modulation are not well understood. Autophagy results from the post-transcriptional downregulation of RHEB expression by *miR-155*. Furthermore, study demonstrated that *miRNA-155* regulates the production of BCG-induced reactive oxygen species via influencing SHIP-1. Research indicates that MTB-infected RAW264.7 cells had significantly increased *miRNA-155* expression, which was closely associated with MTB intracellular survival. However, the *miR-155* gene promotes iNOS production and classical activation (M1 polarization) in alternatively activated (M2) macrophages by targeting CEBPβ. The study demonstrated that *miR-155* stimulates LC3 processing and puncta formation in BCG-challenged and unchallenged RAW264.7 cells. Macrophage autophagy and intracellular microorganism reduction are caused by miR-155. Moreover, Atg7 knockdown or 3-MA autophagy suppression lowered miR-155-mediated autophagy and bacterial mortality. 3-MA also significantly boosted BCG survival after 6 h in control mimic-treated RAW264.7 cells, despite low LC3-II decrease. In addition, 3-MA inhibits nitric oxide generation, boosting mycobacteria survival. Targeting *ATG3* in human dendritic cells allows MTB-induced *miRNA-155* to suppress autophagic processes. Serum *miRNA-155* concentrations in patients with active tuberculosis were markedly elevated compared to those in healthy individuals, suggesting that *miRNA-155* serves as a biomarker for the diagnosis of active tuberculosis. These results illustrate that *miRNA-155* is a crucial regulator of tuberculosis infection by altering host gene expression, either directly or indirectly [10–13]. The human macrophage cell line THP-1, when exposed to the MTB strain H37Rv, exhibited an elevation of *miR-155*. In addition to macrophages, CD14+ cells or peripheral blood mononuclear cells (PBMC) isolated from persons with active tuberculosis exhibited an upregulation of *miR-155*. Concerning the mechanism of *miR-155* immunomodulatory action, previous studies indicated that MTB may target the *ATG3* gene to alter *miR-155* production, thereby inhibiting autophagy in human dendritic cells for its survival (Figure 1) [7,14]. Importantly, the *role of miRNAs-155* is crucial in MTB infection, since one important modulator of innate immunity is *miR-155-5p*. Early on in the infection process, innate immunity is suppressed due to the increased expression of *miR-155-5p* caused by the MTB infection. In this context, the pure protein derivative 10 stimulated PBMCs from active TB patients, increasing the production of *miR-155* and *miR-155×*. Thus, the *miR-155* was thoroughly examined. Though the innate immunological responses brought on by MTB infection and the macrophage apoptosis brought on by Bacillus Calmette–Guérin (BCG) infection were both suppressed by *ESAT6*-dependent *miR-155*. However, *miR-155* targets immune-related proteins like C/EBP and SHIP1, which negatively regulate the IL-6 signalling pathway, along with *SOCS1. ESAT-6* may control *SHIP-1, C/EBP β*, or both via *miR-155* to affect immunological response [15,16].

### 2.2. Role of miR-29 in TB pathogenesis

The recent meta-analysis study observed that *miR-29a* is effective in discriminating latent pulmonary TB from active TB (AUC = 84.35%) and recognizing active TB from healthy patients (AUC = 81.37%). Blood-derived *miR-29a-3p* can identify active TB patients from healthy controls, suggesting it could be a useful biomarker. This miRNA was found in plasma samples, unlike sputum, suggesting its potential use in diagnosing TB, especially in children and extra-pulmonary cases. Also, the results supported earlier opinions. *miR-29a* has 82% sensitivity and 81% specificity for adult tuberculosis diagnosis and 74% and 89% for kid diagnosis [17,18]. Also, multiple studies have shown that the gene expression patterns of macrophages and NK cells differ among active TB patients, latent patients, and healthy controls (HC). In systemic infections produced by *Mycobacterium bovis* or *Listeria monocytogenes*, NK cells downregulate *miR-29*, an mRNA repressor in viral infections targeting the HIV-1 *3′UTR* region, reducing *IFN-* γ. The host’s Listeria resistance is improved by *miR-29a*. Compared to HCs, active pulmonary TB patients had higher serum and sputum *miR-29a* levels. *Mycobacterium avium*-infected human macrophages had increased *miR-29a* and *miR-let-7e*, which targeted caspases 7 and 3. Since Mycobacteria infections reduce apoptosis, miRNAs regulate it. MiRNAs regulate gene expression and cellular composition in TB patients. Many miRNAs regulate T cell development and function. It is also known that miRNAs regulate dendritic, NK, macrophage, and innate immune cell function [19]. Hence, patients with active and latent TB had markedly elevated levels of blood *miR-29a-3p* in comparison to healthy controls. Consequently, *miR-29a-3p* along with *miR-155* play a significant role in prognosis of the disease, might serve as a possible biomarker for pulmonary TB diagnosis (Table 1) [20].

**Table 1.** Functional analysis of miRNA-155 and miR29 linked to tuberculosis.

Study	Types of miRNAs	Role	Clinical outcomes	Technical aspects: in-vivo/in-vitro
Zheng, M. L et al., 2016	*miR-155*	controlling the anti-mycobacterial response through autophagy	good performance between PTB and control groups.	Between PTB and control groups, *miR-155* with high AUC of 0.976. *miR-449a, miR-212*, and *miR-132* with AUC values of 0.947, 0.931, and 0.930.
Ying H et al., 2020	*miR-155*	controlling the anti-mycobacterial response through autophagy	The sensitivity and specificity of *miR-155* identification were 94.1% and 87.7%.	68 PTB and 122 non-PTB patients.
Wang, L. H et al., 2025	*miR-155*	controlling the anti-mycobacterial response through autophagy	The serum *miR-155* AUC-0.968, threshold of 1.50. 91.80% sensitivity. 89.00% specificity. sputum *miR-155* AUC for TB diagnosis was 0.977 with a threshold of 1.70, resulting in 96.40% sensitivity and 90.0% specificity.	PTB-110 cases, and HC-50. In this active TB-50/latent TB-50.
	*miR-146a*	Affecting expression of TNF receptor associated factor-6 (*TRAF-6)*	High AUC predicts better TB diagnosis *miR-146a/155*. Serum *miR-146a*‘s AUC −0.925 cut-off of 1.25, leading in 85.50%-sensitivity 87.00%-specificity. sputum *miR-146a*‘s AUC-0.940, threshold of 1.35, 80.90% sensitivity. 84.55% specificity.	
Ying, H et al., 2020	*miR-155*	controlling the anti-mycobacterial response through autophagy	The *miR-155* detection sensitivity-94.1%. specificity-87.7%. It exceeded anti-TB antibodies and sputum smears.	68 PTB patients and 122 non-PTB
Ndzi, E. N et al., 2019	*hsa-miR-29a-3p*	*miRNA-29a-3p* reduces immunological response by downregulating IFN-γ production in T cells post-transcriptionally.	*miR-29a-3p* was good in distinguishing ATB from LTB and HEC (81.37% and 84.35%, respectively).	A total of 162 cases, active TB-84, latent-35, HC-43
Angria N et al., 2022	*hsa-miR-29a-3p*	*miR-29a-3p* attenuates the immune response by post-transcriptionally downregulating interferon (IFN)-γ production in T cells.	For latent TB, the *miR-29a-3p* ROC curve – AUC of 0.808 (95% CI: 0.698–0.919), 70% specificity, and 84.8% sensitivity. Neither in patients with active or latent pulmonary TB (*R* = 0.005; *p* = 0.62) nor in healthy individuals (*R* = 0.060; *p* = 0.19), did the expression of *miR-29a-3p* show any association with INF-γ levels.	50 active TB, 33 household contacts with positive IFN-γ release, and 30 healthy controls were included in this study.

### 2.3. Circulatory miRNAs roles in diagnosis of TB

Circulating miRNA influences gene expression and immunological and non-immune cell activity. Endogenous regulators include many miRNAs. For several diseases and pathologies, stable circulating miRNAs have been reported, *miR-let-7a, −221, −26a, −191, −320a*, and *-93-5p*. The most often utilized reference genes for normalizing circulating miRNA expression are RNU6B and miR-16. After geometric ranking analysis, *miR-22-3p* was the most stable at 1.19 and 1.73, followed by *miR-93-5p*. *miR-16-5p* was least steady at 2.83. Total stability: *let-7i-5p > let-7a-5p > miR-16-5p > miR-22-3p > miR-93-5p*. Thus, host, environmental, or both variables may affect circulating miRNA stability and gene expression [21]. Also, comparing the circulatory-miRNA (c-miRNA) signature to the *RNA-CoR* (RNA based Correlate of Risk) signature may help c-miRNAs regulate the interferon response to TB. RNA-CoR genes showed a strong correlation with mycobacteria-induced *miR-21*, a marker of immune cell activation. RNA-CoR genes, including *FCGR1B*, were negatively associated with miR-26a, which suppresses the macrophage response to IFN-γ, and *miR-30b*, which suppresses the expression of Fc receptors and the generation of pro-inflammatory cytokines [22]. However, c-miRNA profiles have associated *miR-21* with exosomes, which are small vesicles derived from cells that transport stable short RNAs, including miRNA, suggesting its role as a secreted biomarker of disease. Exosomes serve as conventional immunomodulators by facilitating cell-to-cell communication. The presence of *miR-21* in various exosomes, including those derived from tumours and immune cells, indicates its important role in the regulation of immunological functions [23]. Most notably, persistent bacterial or viral infections increase the expression of *miR-21* in immune-compromised conditions include asthma, psoriasis, cancer, and the development of regulatory immune cells such as Th2, regulatory T-cells, or M2 macrophages, as well as the reprogramming of pro-inflammatory Th1-cells and M1 macrophages, are associated with a number of these disorders. Therefore, damage-associated molecular pattern molecules (DAMPs) or pathogen-associated molecular pattern molecules (PAMPs) dysregulating *miR-21* may create an immunosuppressive, anti-inflammatory milieu that causes illness [24]. Besides that, mycobacterial species survive and thrive in macrophages and create *miR-21* via interfering with host responses. Also, *miR-21* activates inflammatory mediators in non-haematopoietic cells, leading to neoplastic transformation [25].

Hierarchical miRs were found in control and childhood TB groups. Peripheral white blood cells were clustered utilizing 29 miRs expressed differentially in TB children and healthy controls (HC). It is intriguing that 14 candidate miRNAs were chosen for validation from 25 tuberculosis-affected children and 21 HCs. Compared to HCs, TB patients exhibited increased *miR-29* but decreased *miR-155, miR-31, miR-1, miR-146a, miR-125b, miR-150*, and *miR-10a*. Thus, any of them might be a potential initial diagnostic biomarker for diagnosis of TB in children [26]. Furthermore, recent research found that exosomal *miR-130b-3p* and *miR-423-5p*, which boost p65 and Cyclin D1 expression, promote lung cancer (LC) development. The study found high levels of these miRNAs in pleural effusion exosomes but not blood plasma [27,28].

### 2.4. Different miRNAs association with drug-resistant TB

The miRNAs are associated with various function in TB significantly, meanwhile drug-resistant TB needs to be studied. However, a recent study found the substantial results that miRNAs roles in drug-resistant (DR) and drug-susceptible (DS) – TB compared to HC. DR-TB alone showed a substantial decrease in *miR-548 m* and *miR-631*, two of the down-regulated miRNAs. Recent study analysed that DR-TB showed a substantial decrease in *miR-548 m* compared to DS-TB, LTB, and HC (fold change: −1.62, *p* = 0.04, −2.11, *p* < 0.05, −1.94 for HC). The *miR-631* decrease was statistically significant compared to DS-TB (FC: −1.64, *p* = 0.01) and HC (FC: −1.62, *p* = 0.008) but not LTB [29]. Additionally, miRNA assessment for DR-TB treatment monitoring: rifampicin-resistant (RR), multi-drug resistant (MDR), or extensively drug resistant (XDR) strains may have formed if *miR-let-7e-5p* is down-regulated and *miR-328-3p* is increased at sputum conversion T (3–5). The study also found that *miR-20a-3p* up-regulation is connected to MDR resistance. Alongside other clinical and microbiological data, the expression of *miR-let-7e-5p* and *miR-328-3p* may facilitate the early monitoring of DR-TB patients after therapy [30]. The miRNA signature model for DR-TB (*miR-let-7e-5p, miR-223-3p*, and *miR197-3p*) demonstrated 100% diagnostic sensitivity and 75% specificity [31]. Similarly, evidence suggests a relationship between miRNAs and DR-TB, as patients with MDR-TB, DS-TB, and healthy controls showed significant variations in *miR-4433b-5p*, CD44, *miR-424-5p*, F11, *miR-199b-5p*, and *KNG1*; *p* < Blood coagulation requires *KNG1* and human platelet miRNA *miR-199b-5p* [32].

### 2.5. Various types of miRNAs are involved in pathogenesis of TB

Recent studies have found that differently expressed genes (Table 2) were associated with up and down regulation miRNAs are links to pathogenesis of TB. Particularly, the PTB patients exhibited considerably lower *miR-454-3p, miR-15a-5p, miR-590-5p, miR-381*, and *miR-449a* expressions than healthy controls. EPTB patients’ miRNA expression was unaltered. Study also evaluates MTC genotype miRNA expression. *miR-590b-5p*, which was upregulated in zoonotic strains and LAM7TUR, was the sole miRNA with pretreatment and post treatment changes. A miRNA signature investigation of active TB patients discovered that *miR-590-5p* was considerably different from healthy patients. However, *miR-590-5p* plays a clinically crucial in individuals infected with TB. In contrast, the LAM7TUR and Beijing strains had higher *miR-15b-5p* and *miR-21-5p* levels than the control and pretreatment patient groups [33]. This study also found that untreated TB patients infected with Beijing strains had considerably higher *miR-21-5p* expression levels than the control group, contradicting to a recent study that reported decreased expression in cured ones due to anti-TB medications may lower *miR-21-5p* by downregulating host defence. Due to improved inflammation and antimicrobial status, untreated TB patients should express higher *miR-21-5p*. The immune system and inflammation may be compromised by anti-TB medications that suppress *miR-21-5p* expression [33–35]. Besides that, in a preliminary study observed that MTB infection and pulmonary TB pathogenesis depend on *miRNA-146a*. Numerous studies have shown that the C allele in *miRNA-146a’s rs2910164* may decrease mature *miRNA-146a* synthesis, altering the inflammatory process. Significantly, *miR-146a* has lower expression in TB than control group, especially Males with TB exhibited higher levels of *miR-146a* expression (median 0.46; range 0.01–11.59) compared to females (median 0.24; range 0.01–1.3), while the difference was not statistically significant (*p* = 0.061). The *miRNA-146ars2910164* C > G single-nucleotide polymorphism (SNP) may influence the immune response to MTB infection, which could increase the risk of pulmonary tuberculosis. Systemic down-regulation of *miRNA-146a*, a negative regulator of the immune response, may exacerbate TB patients’ inflammatory response. Unlike the mature *miRNA-146a* sequence, *SNP rs2910164 C > G*, encoded on chromosome 5q33, is present in the precursor stem region, + 60 relatives to the first nucleotide [36,37].

**Table 2.** Significant outcomes of various miRNAs in diagnosis of TB.

Study	Types of miRNAs	Clinical outcomes	Technical aspects: in-vivo/in-vitro
Korma W et al., 2020	*miR-16-5p, −22-3p, −93-5p*	Bestkeeper models for miRNA expression data assessment show acceptable standard deviations (below 1) for *miR-93-5p, miR-22-3p, and miR-16-5p. miR-22-3p and miR-16-5p* exhibited 0.90 and 0.94 standard deviations, whereas *miR-93-5p* was the most stable at 0.82.	68 trial participants (26 HC, 23 LTBI, and 19 TB) were included in this study
Zhou M et al., 2016	*miR-146a, miR-10a, miR-125b, miR-150, miR-29, miR-1, miR-155, −31*	Eight miR’s demonstrated excellent diagnostic value with AUCs of 0.996 (95% CI, 0.914–1.0). Both sensitivity and specificity are 95.8% and 100%. *miR-1, miR-155, miR-31, miR-146a, miR-10a, miR-125b, miR-150*, and *miR-29* suggested as early diagnostic biomarker.	Total of 129 childrens were enrolled. 28 TB and 24 HC
Ashirbekov Y et al., 2024	*miR-376a-3p, miR-15b-5p*	Compared to controls, TB patients had much greater and lower *miR-376a-3p* and *miR-15b-5p* levels.	38 pulmonary TB patients, 41 healthy controls, and 68 LC patients
Kang, H J et al., 2024	*Tuberculous exosomes, miR-130b-3p and miR-423-5p*	PCR sequencing in cell studies and nude mice trials showed that tuberculous exosome-treated A549 cells had much greater *miR-130b-3p* and *miR-423-5p* levels.	Male BALB/c mice, 12 mice got a subcutaneous transplant of 2 × 10^6^ A549 cells in their flanks.
Buha I et al., 2022	*miRNA-146a*	Compared to controls, TB patients showed significantly lower *miRNA-146a* expression (median 0.30, 0.01–1.72). *p* < 0.001 vs. median 1.000 (0.06–11.59).	TB patients − 44, HC-17.
Sun X et al., 2022	*miR-378*	ATB patients express more *miR-378* than LTBI ones. serum *miR-378* > 1.490 was sensitive and specific for TB diagnosis. In TB patients, IFN-γ, TNF-α, and IL-12 levels were low, whereas IL-4, IL-6, and IL-1β levels were increased. In the active group, serum miR-378 levels were favourably connected with IL-4, IL-6, and IL-1β, while IFN-γ, TNF-α, and IL-12 concentrations were adversely correlated	A total of 126 TB patients (63 active and 63 latent) and 62 HC were selected.
Liu J et al., 2023	*let-7d-5p, miR-140-5p*	Panel ROCs for biomarkers. (a) Training ROC curve AUC was 0.93; (b) Test was 0.923. LOOCV’s ROC results showed that our *let-7d-5p* and *miR-140-5p*-trained model could differentiate LTBI from ATB or HC with an AUC > 0.9.	GSE25435 found 174 DEMs between ATB and LTBI, including 39 downregulated and 51 upregulated miR’s. The GSE29190 dataset comprised 24 DEMs
Sampath P et al., 2023	*miR-548 m, miR-631*	DR-TB alone decreased *miR-548 m* and *miR-631* more than any other group. The decrease in miR-548 m in DR-TB was significantly different from DS-TB, LTB, and HC (FC: −1.94, *p* < 0.05 vs. FC: −1.62, *p* = 0.04). However, *miR-631* declined significantly compared to DS-TB (FC: −1.64, *p* = 0.01), HC (FC: −1.62, *p* = 0.008), but not LTB.	(DS-TB) (*n* = 6), latent TB (LTB) (*n* = 6), and drug-resistant TB (*n* = 6), and HC-6 were included in this study.
Carranza C et al., 2021	*let-7e-5p, −197-3p, and −223-3p*	The DR-TB (AUC = 0.96, 95%, CI = 0.907–1) and MDR-TB (AUC = 0.95, 95%, CI = 0.89–1) groups showed greater sensitivity in separating HC from those with TB when *miR-let-7e-5p, −197-3p*, and *-223-3p* were used.	Extracellular Vesicles were recovered from serum of 26 DR-TB patients and 16 HC.
Cui X et al., 2025	*exo-miR-766-3p*	Exosomal *miR-766–3p, miR-376c-3p, miR-1283*, and *miR-125a-5p* had AUCs of 0.8963, 0.8313, 0.8125, and 0.8050, respectively. Notably, Plasma-derived *exo-miR-766-3p* lowered *NRAMP1* expression in A549 cells, while *miR-766-3p* inhibition enhanced it.	The study included 32 TB patients and 32 HC.

A recent study analysed serum samples from patients with active and latent tuberculosis, revealing elevated levels of *miR-378* compared to healthy individuals, with the active group exhibiting the highest expression levels. In TB patients, *miR-378* was always raised. Also, this study found that *miR-378* had an AUC of 0.767, a cut-off value of 2.130, 92.060% sensitivity, and 52.380% specificity for distinguishing active and latent groups. Thus, this study suggests *miR-378* may be a TB biomarker [38]. On the other hand, the roles of *let-7d-5p* and *miR-140-5p* in the pathophysiology of TB remain unclear, and a recent study found that their expression was down-regulated in patient serum when compared to healthy controls. In this context, the biological importance of their target genes was concentrated in kinase activity regulation, vascular development, and growth factor response. However, miRTarBase and miRDB generated 86,781 paired miRNA-target gene interactions with 2459 miRNAs and 8849 target genes. By blocking translation in target genes’ 3’ untranslated regions (3’UTRs), microRNAs downregulate them. After eliminating miRNAs that upregulate mRNAs, ATB and LTBI had 2156 target genes regulated by 89 miRNAs while LTBI and HC had 2139 by 81 miRNAs. Training set AUC for the biomarker panel was 0.930 (sensitivity = 100%, specificity = 88.5%) and test set AUC was 0.923 (sensitivity = 100%, specificity = 92.3%). The receiver operating characteristics (ROC) of LOOCV data showed that *let-7d-5p* and *miR-140-5p*-trained models could distinguish LTBI from ATB or HC with an AUC greater than 0.9. Thus, *let-7d-5p* and *miR-140-5p* are essential TB indicators. In addition, Dong et al. found that *miR-140-5p* reduced lung fibrosis via blocking the Wnt1/β-catenin pathway [39,40].

Recent study on validation of Solexa sequencing research found significant differences in 181 serum miRNAs between 10 TB patients and 10 healthy controls (fold change > 2, *p*< 0.05). A total of 88 miRNAs were down-regulated (<0.50-fold, *p* < 0.05) and 93 were up-regulated (>2.0-fold, *p* < 0.05). Solexa found 19 autophagosome maturation miRNAs with above 10 copies for qRT-PCR validation. Study also investigated 11 miRNAs with fold changes (TB patients/HCs) ≥1.2 or ≤0.8 and *p* < 0.05 from the training set. Sixty TB patients and 41 healthy controls had 10 miRNAs. Patients with TB showed substantially higher levels of *miR-17-5p, −20b-5p, −378a-3p*, and *-423-5p* (*p* < 0.001). In addition to TB patients’ serum, PBMCs the primary source of macrophages that remove MTB, also showed increased *miR-423-5p* [41]. Also, in the preliminary screening study examined *miR-181b-5* connection. Using qRT-PCR, plasma from eight HC and eight TB patients was quantified for *hsa-miR-181b-5p*. In TB patients’ plasma samples, *miR-181b-5p* expression increased significantly. Also, in monocytes from whole blood samples, infection with MTB with RAW 264.7 and BMDM in mouse model entails the regulation of *SAMD9L* expression by TLR2 and HIF-1. The analysis revealed that *miR-181b-5p* and *SAMD9L* do not interact, despite being a valuable TB marker [42]. Also, multiple studies have shown that in mycobacteria-infected macrophages, *miR-17-5p* acts as an immunomodulatory regulator of autophagy. *Hsa-miR-17-5p* regulates autophagy in MTB-infected macrophages via targeting *Mcl-1* and *STAT3* [43]. The preferred pathways were systematically categorised and organised. The inhibition of FOXO1 has been demonstrated to inhibit apoptosis and the inflammatory response. Interleukin-6 demonstrates pro-inflammatory characteristics. The suppression of the Egr1/TGF-β/Smad pathway by miRNA-181a-5p is associated with the regulation of cell growth [44–46].

Various differentially expressed genes (DEGs) play a vital role in disease pathogenesis. Likely, abnormal levels of *miR-191, miR-146a, miR-20, miR-22*, and *miR-320* are among the patterns of miRNA expression in body fluids that are linked to the severity of the disease and may be signs of active tuberculosis rather than latent infection [47]. The *miR-15/16* family member *miR-15a-5p* modulates signalling pathways involved in apoptosis, cell cycle regulation, and inflammatory cytokines. Also, *miR-15a-5p* regulates pro- and anti-inflammatory responses by targeting genes such *BCL2, IKKα*, and NF-κB signalling cascade components. *miR-15a-5p* can regulate inflammation and macrophage-mediated bacterial clearance, making it a potential TB biomarker and treatment target [48–51]. Notably, silencing endogenous *miRNA-31* can inhibit *PD-1/PD-L1* expression on lung CD4+/CD8+T cells, increase MTB burdens, and worsen lung pathology, suggesting a novel TB mechanism [52]. Notably, targeting the 3′ UTR of the TNF mRNA, the miR-125b was up-regulated in MTB-stimulated macrophages, reducing TNF production. Additionally, another study indicated that following TB infection, *miR-125b-5p* is significantly expressed in a variety of immune cells, mostly monocytes-macrophages. In particular, *miR-125b* decreases inflammatory responses via TNF-α [24,53,54].

A recent study used qPCR to assess the plasma biomarker potential of 10 miRNAs and 9 proteins. The AUC for all 19 analytes upon diagnosis was 1.000, indicating 100% sensitivity and 100% specificity in TB patients. The panel was reduced to five miRNAs and proteins (IP-10, *miR-29a, miR-146a, miR-99b*, and *miR-221*), yielding an AUC of 0.995, sensitivity of 96%, and specificity of 97%. IP-10 was the most effective TB biomarker among 19 plasma miRNAs and proteins. Results reveal that a specific mix of miRNA and protein biomarkers may enhance protein or miRNA biomarker candidates to achieve WHO’s criteria for elevated sensitivity and specificity need several biomarkers. In serum exosomes of TB patients, *miRNA-146* expression was significantly elevated compared to the healthy cohort. Additionally, significant increases were noted in the expression levels of *miR-425, miR-484*, and *miR-96*. Subsequently, exosomes are crucial for cellular communication, and diseases cause abnormalities in the exosomal process. Exosomal miRNAs have also been suggested by numerous researchers to be a marker of the illness progression, exosomes show promise as therapeutic agents and biomarkers for diagnosis [55–59]. Downregulation of *miR-146a-5p* following MTB infection enhanced the expression of UBE2L6, which functions as a ubiquitin and ISG15 E2 enzyme. UBE2L6 and miR-146a-5p appear to serve as biomarkers for tuberculosis. The role of UBE2L6 in tuberculosis is significant [60–62].

Additionally, the network’s miRNA *hsa-miR-185-5p* was 3.65 times more abundant in the cured TB group than in the untreated TB group. In addition, in a case-control study of HIV-negative TB patients, *miR-215* was significantly higher after 2 months of medication compared to untreated patients. This study confirmed that the downregulated *hsa-miR-215-5p* likely affects the TGF-α signalling pathway, as previously reported [29,63,64]. Interestingly, nicotine decreases macrophage phagocytosis. However, *miR-296-3p* targets *SIRPα* and reduces its mRNA and protein levels in macrophages by binding to two locations in the *3′-UTR*. It is interesting that *miR-296-3p* inhibits nicotine from boosting *SIRPα* mRNA and protein levels, indicating its importance in managing the interaction between SIRPα and nicotine in macrophages [65,66].

### 2.6. Various differentially expressed genes association with prognosis of TB

The regulation of gene expression is additionally affected by other principal categories of long noncoding RNAs (lncRNAs), small noncoding RNAs (sncRNA), such as small nucleolar RNAs (snoRNA), small nuclear RNAs (snRNA), and PIWI-interacting RNAs (piRNA) [67]. In this context, recent study used CNC to analyse mRNAs and lncRNAs. In TB, differentially expressed lncRNAs target *IL6ST, IL18, BCL2, IL5, TLR6*, and *NOD2*. Toll-like receptors (TLRs) identify MTB and its cells, activating the macrophage system and triggering acquired and innate immunity. Aberrant lncRNA expression affects both the pathogenic process of TB and lncRNA polymorphisms linked to susceptibility, demonstrating that lncRNAs play a major role in its pathogenesis. Besides that, in the therapy of TB, lncRNAs are essential. Because the gut microbiota controls lncRNAs to have immune-protective effects against TB, research suggests that the gut-lung axis may be a novel therapeutic target for pulmonary disorders [68–71]. In addition, circRNA functions are still mostly unknown. CircRNAs modify the expression of parental genes and can be produced from exonic or intronic sequences. Four circRNAs of interest were sequenced in the current investigation after microarray screening. Among these, active TB plasma showed up-regulation of *hsa_circ_091692, hsa_circ_102295*, and *hsa_circ_102295* [72,73]. Notably, a preliminary investigation of c-miRNAs in active PTB patients showed 33 downregulated and 59 elevated signatures compared to non-TB controls. This study examined EPTB’s most upregulated and downregulated *miRNAs, hsa-miR-425-5p* and *hsa-miR-4523* [74,75].

## 3. Conclusion

This review analysed the diverse functions of miRNAs in MTB and their role in the pathogenesis of the disease. However, miRNAs, also known as epigenetic modulators, regulate post-transcriptional gene expression in many physiologic and pathological processes [76]. Also, the relationship between *let-7b-5p* and *miR-30a-3p* and *IL-27* and *CXCL9/10/11*, as well as their expression in TB patients’ lesion tissue and peripheral blood [77], provides a foundation and new ideas for further research in DS and DR-TB.

## Data Availability

The authors confirm that the data supporting the findings of this study are available with in the article, any further underlying data will be made available upon reasonable request.
